# Impact of self-esteem and sex on stress reactions

**DOI:** 10.1038/s41598-017-17485-w

**Published:** 2017-12-08

**Authors:** Lydia Kogler, Eva-Maria Seidel, Hannah Metzler, Hanna Thaler, Roland N. Boubela, Jens C. Pruessner, Ilse Kryspin-Exner, Ruben C. Gur, Christian Windischberger, Ewald Moser, Ute Habel, Birgit Derntl

**Affiliations:** 1 Department of Psychiatry and Psychotherapy, Medical School, University of Tübingen, Tübingen, Germany; 2 Social, Cognitive and Affective Neuroscience Unit, University of Vienna, Vienna, Austria; 3 Department of Applied Psychology: Health, Development, Enhancement and Intervention, Faculty of Psychology, University of Vienna, Vienna, Austria; 4 Département d’Etudes Cognitives, Ecole Normale Supérieure de Paris, Paris, France; 5 Max Planck Institute for Psychiatry, Munich, Germany; 6 MR Center of Excellence, Medical University of Vienna, Vienna, Austria; 7 Institute for Psychology, University of Konstanz, Konstanz, Germany; 8 Neuropsychiatry Division, Department of Psychiatry, University of Pennsylvania, 19104-4283 Philadelphia, PA USA; 9 Centre for Medical Physics and Biomedical Engineering, Medical University, 1090 Vienna, Austria; 10 Department of Psychiatry, Psychotherapy and Psychosomatics, RWTH Aachen University, Pauwelsstrasse 30, 52074 Aachen, Germany; 11 JARA BRAIN Institute I, Translational Brain Medicine, Jülich/Aachen, Germany; 12 Center for Integrative Neuroscience, University of Tübingen, Tübingen, Germany; 13 LEAD Graduate Training and Research Center, University of Tübingen, Tübingen, Germany

## Abstract

Positive self-evaluation is a major psychological resource modulating stress coping behavior. Sex differences have been reported in self-esteem as well as stress reactions, but so far their interactions have not been investigated. Therefore, we investigated sex-specific associations of self-esteem and stress reaction on behavioral, hormonal and neural levels. We applied a commonly used fMRI-stress task in 80 healthy participants. Men compared to women showed higher activation during stress in hippocampus, precuneus, superior temporal gyrus (STG) and insula. Furthermore, men outperformed women in the stress task and had higher cortisol and testosterone levels than women after stress. Self-esteem had an impact on precuneus, insula and STG activation during stress across the whole group. During stress, men recruit regions associated with emotion and stress regulation, self-referential processing and cognitive control more strongly than women. Self-esteem affects stress processing, however in a sex-independent fashion: participants with lower self-esteem show higher activation of regions involved in emotion and stress regulation, self-referential processing and cognitive control. Taken together, our data suggest that men are more engaged during the applied stress task. Across women and men, lower self-esteem increases the effort in emotion and stress processing and cognitive control, possibly leading to self-related thoughts in stressful situations.

## Introduction

Self-esteem is the value we place on ourselves, with high levels associated with a positive self-evaluation and a positive self-attitude^1,2^. It constitutes an important psychological resource: High levels of self-esteem are protective against adverse mental health outcomes and are considered an important resilience factor^3,4^. Contrarily, low levels of self-esteem are attributes of nearly all psychiatric diagnoses^5^, particularly major depression and eating disorders. Meta-analyses on self-esteem consistently reveal sex differences, with men reporting higher global self-esteem than women^6,7^. An inferior positive self-evaluation in women may contribute to the well-known female preponderance for specific psychiatric disorders, such as depression, eating disorders or anxiety disorders^8,9^. The neural basis of self-esteem has been explored in a variety of affective neuroimaging tasks and includes areas involved in cognitive control, affective and self-referential processing, such as lateral and medial frontal cortical areas, anterior and posterior cingulate gyrus (ACC, PCC), superior temporal areas, anterior insula and precuneus in mixed samples^10,11^. For an implicit self-esteem task, sex differences were reported in medial and lateral frontal areas^2^.

Self-esteem as a psychological resource may enable subjects to cope more effectively with stressful events^3^. Stress is triggered by situational demands that exceed the cognitive resources of an individual^12^ and the attitude toward coping with such situations may influence the individual’s stress reaction. Indeed, the subjective stress reaction is related to individual self-esteem: Lower scores are associated with inferior performance and heightened cortisol reactions to achievement stress and challenging situations^13,14^. Contrarily, high levels of psychological resources (including self-esteem) have been linked to lower reactivity to stressful events^3,15^.

Several studies on acute stress reactions investigated the impact of sex, most frequently on stress-related cortisol responses, with some studies observing significant increases post stress in men as compared to women^16–18^. Neuroimaging studies on acute stress show an increase in neural activation in e.g., the dorsomedial (DMPFC) and dorsolateral prefrontal cortex (DLPFC), inferior frontal gyrus (IFG), ACC, insula, superior temporal gyrus (STG), striatum and precuneus^19–21^. The few studies that investigated sex differences revealed increased activation in the right STG, right insula, PCC, bilateral ventral striatum as well as the amygdala in women and in the right anterior DLPFC, and the putamen in men during stress^22,23^.

Notably, neural areas associated with self-esteem^2,10,11^ widely overlap with regions of the stress network^19–21^. For some of these areas, such as the amygdala, the anterior insula, lateral prefrontal areas and the PCC, sex differences during stress processing have also been reported^22,23^. It seems that women preferentially recruit regions involved in self-referential and affective processing, which are also more negatively associated with self-esteem^10^, whereas men activate regions associated with cognitive regulation, which correlate positively with self-esteem^11^. Thus on a neural level, prior results suggest sex-specific associations between self-esteem and stress reaction. Moreover, these results imply that beneficial effects of self-esteem on stress coping behavior may differ between women and men. However, to the best of our knowledge no study has investigated these associations from a sex and self-esteem perspective. The aim of the present study was to examine whether self-esteem distinctively affects the behavioral, hormonal, and neural stress reaction in women and men. We were particularly interested in activation of the right insula and the right STG as both areas have been reported to show sex-specific effects during stress processing and for self-esteem^2,22^.

Based on previous findings, we hypothesized that women and men differ in their self-esteem, with men showing higher scores. Furthermore, we expected lower levels of self-esteem to be associated with higher activity in regions associated with affective and self-referential processing (insula). Contrarily, we expected higher self-esteem to go along with a stronger activation in regions of attention and cognitive control (STG).

## Results

Eighty healthy participants (40 females) aged 19 to 35 years were included in the study. Half of the women were measured during their early follicular phase, the other half during their mid-luteal menstrual cycle phase. Participants performed a list of neuropsychological tests and filled out several questionnaires. Most importantly, all participants performed a modified version of the Montreal Imaging Stress Task (MIST)^19^ to induce achievement stress. As in the original version, participants were required to solve arithmetic problems. Modification addressed the social aspects, which were excluded in this version: participants did not receive negative social feedback between runs and did not see their performance in comparison to performance of a peer-group. Additionally, we assessed hormone concentration of cortisol, testosterone and progesterone (pre- and post-stress) using saliva samples.

### Sample characteristics

Women and men did not differ in age (p = 0.710), intelligence (p = 0.833), stress coping strategies (CISS; all p-values ≥ 0.070) or achievement motivation (AMI; p = 0.823).

### Subjective ratings

Women and men did not differ in self-esteem (p = 0.228; women: mean (M) = 39.25, standard deviation (STD) = 4.6, men: M = 40.58, STD = 5.1).

The 2 (sex) by 2 (time) ANOVA on the positive mood ratings (using the Positive and Negative Affect Scale, PANAS^24^) showed a significant time effect (F(1,77) = 5.136, p = 0.026, ηp^2^ = 0.063) with a decrease in positive mood from pre- to post-stress. All other main effects or interactions were non-significant (all p-values ≥ 0.578). The 2 (sex) by 2 (time) ANOVA on the PANAS negative mood ratings showed a significant time effect (F(1,77) = 10.424, p = 0.002, ηp^2^ = 0.119) with an increase in negative mood from pre- to post-stress. All other main effects or interactions were non-significant (all p-values ≥ 0.203).

Regarding the emotional self-rating of basic emotions (ESR^25^), the 6 (emotion) by 2 (sex) by 2 (time) ANOVA showed a significant emotion effect (F(5,390) = 52.958, p < 0.001, ηp^2^ = 0.404) and a significant emotion-by-time interaction (F(5,390) = 15.755, p < 0.001, ηp^2^ = 0.168). No further main effect or interaction reached significance (all p-values ≥ 0.257). Disentangling the significant emotion by time interaction revealed a significant increase in anger and disgust ratings post-stress (p < 0.001) while no significant changes occurred for the other emotions (all p-values ≥ 0.199).

### Task performance

Due to technical problems, behavioral data from three male participants are missing from the analysis. Analysis of sex and condition effects on error ratio revealed a significant sex effect (F(1,75) = 21.542, p < 0.001, ηp^2^ = 0.223), with higher error ratios in women than men, i.e. men made fewer errors. No condition effect (F(1,75) = 0.196, p = 0.659) and no significant interaction (F(1,75) = 0.368, p = 0.546) occurred.

### Hormones

#### Cortisol

Three subjects had to be excluded due to outlying cortisol data (M+/− 2 STD; 1 woman). The 2 (sex) by 2 (time) ANOVA showed a significant main effect of time (F(1,75) = 7.893, p = 0.006, ηp^2^ = 0.095), with a decrease in cortisol levels after the task. Furthermore, a significant sex-by-time interaction emerged (F(1,75) = 6.291, p = 0.014, ηp^2^ = 0.077): Women and men had comparable cortisol levels before the task (p = 0.882), but men showed higher cortisol levels compared to women after the stress task (p = 0.012) (Table 1), i.e., cortisol decreased in women after the task but remained stable in men. No significant main effect of sex occurred (p = 0.153).

**Table 1:** Mean (standard deviation) of cortisol, testosterone and progesterone (pg/ml, log-transformed) before (pre-stress) and 25 minutes after stress onset (post-stress) as well as p-values for the comparison (pre vs. post) are listed.

	Women (n = 40)			Men (n = 40)		
	Pre-stress	Post-stress	Pre vs. Post (p-value)	Pre-stress	Post-stress	Pre vs. Post (p-value)
Cortisol	3.35^a^ (0.20)	3.23^a^ (0.20)	<0.001	3.35^b^ (0.18)	3.34^b^ (0.19)	0.839
Testosterone	1.21 (0.37)	1.16 (0.37)	0.122	1.83 (0.27)	1.91 (0.21)	0.009
Progesterone	1.73^a^ (0.38)	1.63^a^ (0.32)	0.009	1.44^b^ (0.32)	1.38^b^ (0.27)	0.333

Note. ^a^n = 39, ^b^n = 38.

#### Testosterone

The 2 (sex) by 2 (time) ANOVA revealed a significant main effect of sex (F(1,78) = 108.264, p < 0.001, ηp^2^ = 0.581), with higher levels in men than in women, no significant time effect (F(1,78) = 0.362, p = 0.549), but a sex-by-time interaction (F(1,78) = 8.998, p = 0.004, ηp^2^ = 0.103), with a significant testosterone increase after stress in men (p = 0.009) but no change in women (p = 0.122) (Table 1).

#### Progesterone

Two subjects had to be excluded due to outlying hormone data (M + /− 2 STD, 1 woman) and data of one male participant was not available. The 2 (sex) by 2 (time) ANOVA revealed a significant main effect of sex (F(1,75) = 16.347, p < 0.001, ηp^2^ = 0.179), with higher progesterone levels in women than in men (Table 1), and of time (F(1,75) = 5.681, p = 0.020, ηp^2^ = 0.070) with higher levels before than after the stress task. The sex-by-time interaction was not significant (p = 0.444).

### FMRI data

#### Whole group

To account for sex differences that emerged on a behavioral level, error rate was included as covariate in the second-level analysis. The contrast of stress > control in the full sample revealed a large cluster comprising right middle and inferior frontal gyri (MFG, IFG), STG and middle temporal gyrus, precentral gyrus, posterior medial frontal area, supramarginal gyrus, right precuneus and cuneus and left middle occipital gyrus. The contrast control > stress revealed a cluster in the bilateral medial frontal areas (including left rectal gyrus, superior orbital gyrus, right caudate nucleus, ACC), the right STG extending to the insula, left MFG, right postcentral gyrus and bilateral angular gyrus.

#### Sex differences

**Figure 1:**
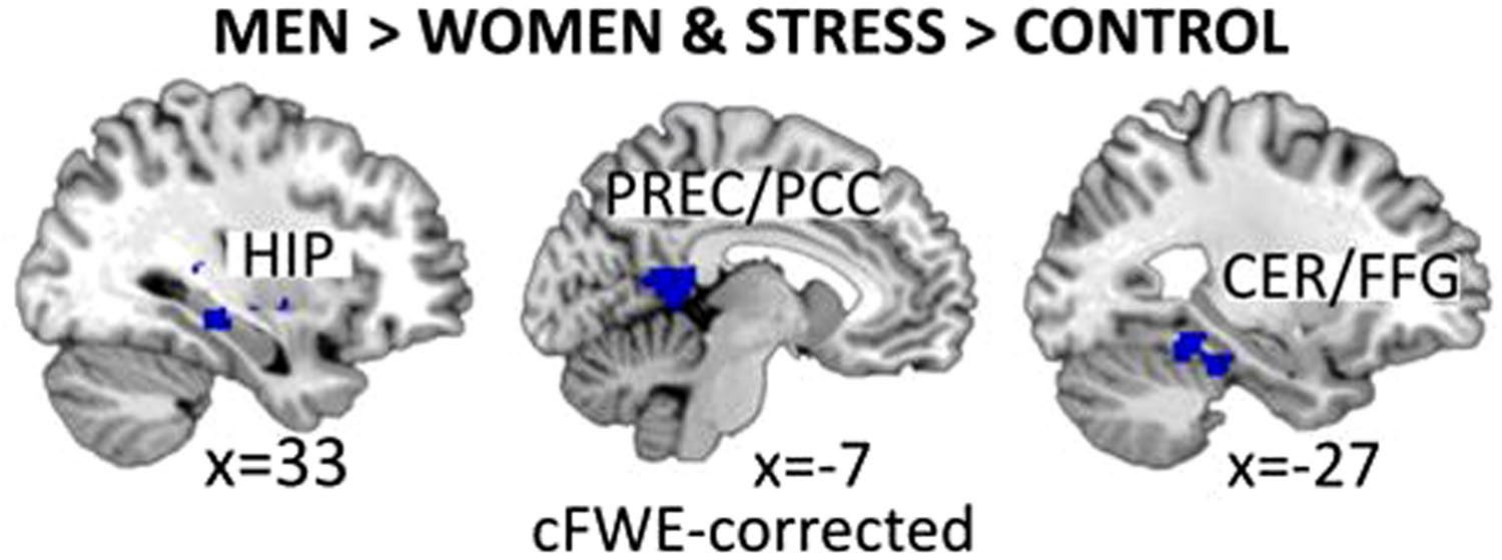
Sex-differences on whole-brain level: Higher activation in men than in women in the stress compared to the control condition. HIP = hippocampus; PREC/PCC = precuneus/posterior cingulate gyrus; CER/FFG = cerebellum/fusiform gyrus.

Directly comparing stress and control condition, men showed significantly stronger activation of the right hippocampus extending to the STG, the left cerebellum extending to the fusiform gyrus and the left precuneus extending to the PCC, than women (Fig. 1). The opposite t-contrast (women > men & stress > control) revealed no significant activation. Full details on cluster size, coordinates and statistical values are given in Table 2.

**Table 2:** Significant clusters in neural activation for specific contrasts. Coordinates are given in MNI space. Only the maximum peak is reported for each cluster. Data is cluster-level FWE-corrected (p < 0.05) and p-values are listed.

Contrast	X	Y	Z	t-value	k	p-value (FWE cluster corr.)	Region
STRESS > CONTROL	48	−64	4	19.4	74077	p < 0.001	R. Middle and temporal gyri, R. Supramarginal gyrus, R. Precuneus, R. IFG, L. Middle occipital gyrus, R. Middle frontal gyrus, R. SMA, R. Precentral gyrus
CONTROL > STRESS	−2	38	−22	8.55	2573	p < 0.001	L. Rectal gyrus
	54	−12	4	7.52	1214	p < 0.001	R. Superior temporal gyrus
	−40	10	50	5.13	673	p < 0.001	L. Middle frontal gyrus
	−42	−78	36	4.94	509	p = 0.001	L. Middle occipital gyrus
	50	−20	60	4.96	466	p = 0.001	R. Postcentral Gyrus
	56	−70	30	5.57	297	p = 0.011	R. Angular Gyrus
STRESS > CONTROL Men > Women	26	−34	0	4.59	446	p = 0.001	R. Hippocampus/STG
	−38	−44	−30	4.69	291	p = 0.012	L. Cerebellum/FFG
	−10	−46	8	4.26	259	p = 0.019	L. Precuneus/PCC
STRESS > CONTROL Women > Men	—	—	—	—	—		—

Note. R = right, L = left, k = cluster size in voxel.

We further performed regression analyses for the significant clusters revealing sex-differences in the whole brain analysis. No association with self-esteem appeared for hippocampus and the cluster cerebellum/fusiform gyrus. For the left precuneus, we observed an effect of self-esteem: The linear regression revealed a significant effect of sex (model R^2^ = 0.147; β = 0.331, t = 3.118, p = 0.003), with higher precuneus activation in men than in women and a significant effect of self-esteem (β = −0.244, t = −2.300, p = 0.024) indicating lower activation in participants reporting higher self-esteem.

### Regression analyses with ROI activation, hormones and self esteem

#### Right insula

For right insula activation we observed a significant sex effect (model R2 = 0.292; β = 5.091, t = 2.728, p = 0.008), with higher activation in men than in women, an influence of self-esteem (β = −1.574, t = −2.659, p = 0.010), with higher self-esteem being associated with lower activation in the right insula during stress (see Fig. 1 for the association between self-esteem and ROI brain activation) and an effect of testosterone (β = −3.520, t = −2.309, p = 0.024), with higher levels of testosterone going along with higher insula activation. Furthermore, interactions of sex-by-self-esteem (β = −3.319, t = −2.130, p = 0.037), sex-by-testosterone (β = −1.323, t = −2.081, p = 0.041) and self-esteem-by-testosterone (β = 3.685, t = 2.252, p = 0.027) emerged but did not survive correction (p > 0.025).

#### Right STG

**Figure 2:**
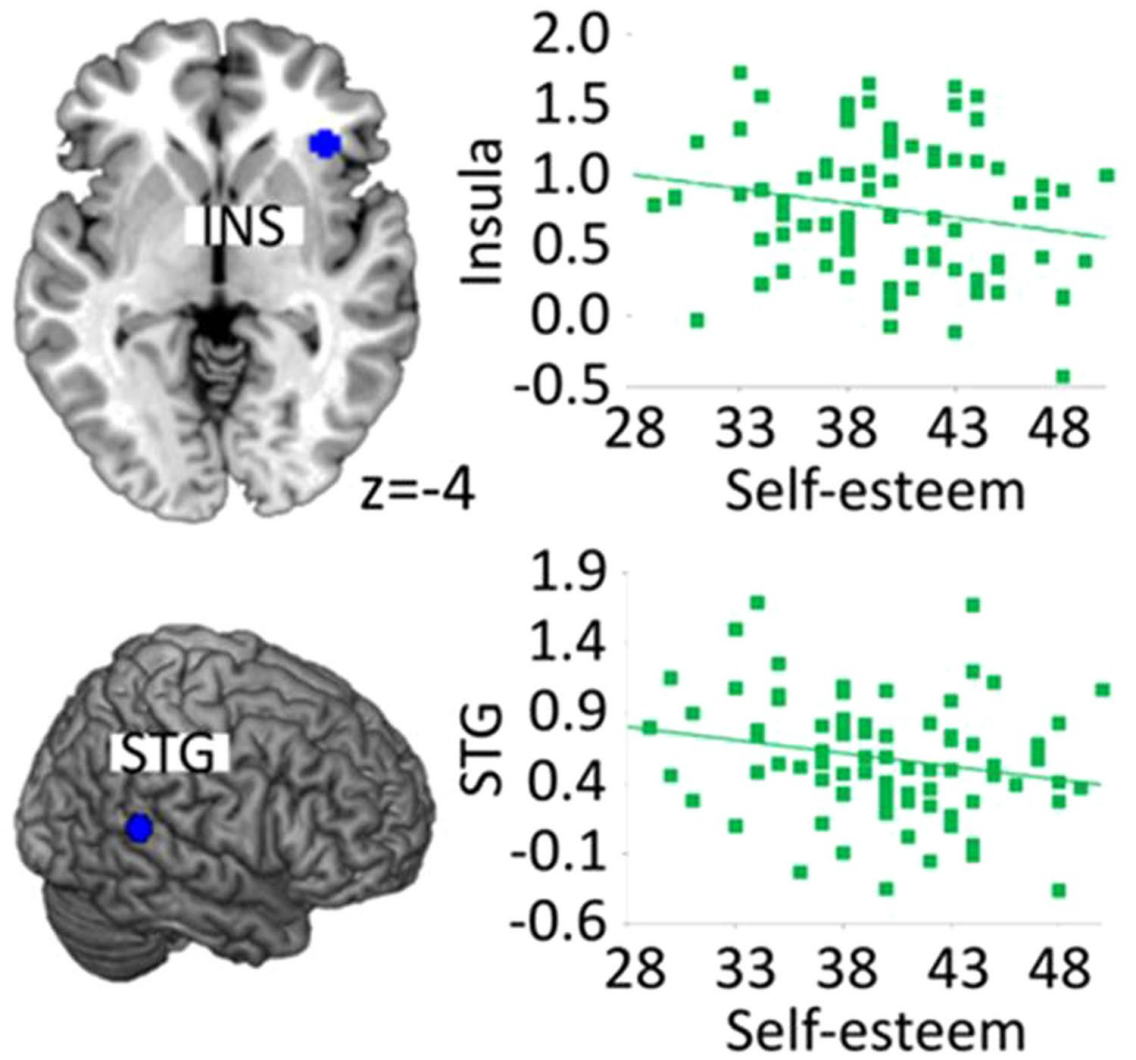
Regression analyses with self-esteem (predictor) and ROI brain activation (outcome). INS = insula, STG = superior temporal gyrus.

For the right STG we observed a significant sex effect (model R2 = 0.205; β = 4.977, t = 2.517, p = 0.014), with higher activation in men than in women, an influence of self-esteem (β = −1.609, t = −2.564, p = 0.012), with higher self-esteem being associated with lower activation in the right insula during stress (see Fig. 1 for the association between self-esteem and ROI brain activation) and an effect of testosterone (β = −3.679, t = −2.277, p = 0.026) with higher levels of testosterone being associated to higher STG activation during stress. Furthermore, the interaction self-esteem-by-testosterone (β = 3.783, t = 2.181, p = 0.032) was significant. However, the later findings did not survive correction (p > 0.025). Please see Fig. 2 for illustration of significant regression analyses.

No significant effects were observed with progesterone or cortisol (all p-values ≥ 0.071).

## Discussion

The aim of this study was to investigate the association of sex and self-esteem on subjective, hormonal and neural stress reactions. Self-esteem was assessed in 80 healthy female and male students who performed a modified version of the MIST-task^19^ by solving mental arithmetic problems under time-pressure. Our data revealed no sex differences in self-esteem scores or in subjective ratings following the stress task. The whole sample showed significant decreases in positive mood and increases in negative mood as well as anger and disgust ratings after the task. In response to stress, male participants showed a smaller cortisol decline, a more pronounced testosterone reaction as well as a greater neural activation with increases in regions that were previously associated with stress. Furthermore, self-esteem had an impact on neural activation during stress.

### Sex differences in stress reactions

#### Subjective ratings

Both male and female participants reported negative emotional reactions to the task: Positive affect decreased and negative affect increased after stress. The lack of sex difference is in accordance with previous results^23,26^, showing sex differences in physiological but not in subjective responses. It has been speculated^27^ that biological processes, rather than subjective perceptions mediate sex differences in response to challenging situations. This claim is supported by our mood, hormone and imaging results.

#### Physiological response

The lack of cortisol increase for the whole group after applying the modified MIST-task is in accordance with previous reports^28,29^. Nevertheless, in our sample men showed a smaller decline in cortisol levels in response to the stress task, which – given the normal decline of cortisol levels over the course of the afternoon – can be interpreted as a mild activation of the HPA axis, in line with previous literature^16,26,27,30^. One might argue that the smaller cortisol change in our male participants reflects feelings of distress and overload. However, cortisol also serves to recruit energy resources and increases vigilance in challenging situations. The latter interpretation for the cortisol dynamics in men seems more plausible since the male-specific testosterone increase indicates active task-focused coping^31^. The observed testosterone increase following the experimental stress induction in male participants is consistent with previous results using the Trier Social Stress Task (TSST)^32^. However, one other study^26^ did not observe testosterone changes using the TSST. Differences in time of testing (late morning) as well as less restrictions concerning food consumption present in the latter experiment^26^ may not allow for direct comparison.

The assumption of an active coping style in men is supported by the superior performance of male compared to female participants, i.e. fewer errors made across items processed. As the task was adaptively adjusting to participants’ performance, confounding baseline differences in arithmetic abilities are rather unlikely. However, a certain degree of difficulty has to be maintained in order for the task to pose a cognitive challenge. Therefore, we assume that men were engaged more intensively in solving the math problems, potentially affecting cortisol and testosterone changes.

It has to be stated that we informed all our participants before inclusion that we are interested in sex differences in math abilities. This may have triggered a selection bias and support gender stereotypical thinking (“men are better in math”). Moreover, we have to critically state that we did not assess participant’s attitude towards mental arithmetics or mathematics, probably contributing to the observed sex differences. Stereotypes that girls and women lack mathematical ability persist, despite mounting evidence of gender similarities in math achievement^33–36^. However, men report more positive math attitudes and affect than women^36^. Therefore, it is up to future studies to investigate whether the observed effects also appear a) when gender stereotype threat is minimized and b) when tasks that favor women, such as verbal memory and social cognition tasks^37^, are used.

#### Neural activation

A network of brain regions including hippocampus, left precuneus, left cerebellum/fusiform gyrus, right anterior insula, right IFG, right STG and right MFG responded to the achievement stress task more strongly in men compared to women. Activation of this network has frequently been reported when participants were confronted with similar mental arithmetic problems using mixed^20,38,39^ or male-only samples^29^. Notably, we previously reported higher STG activation in women than in men during stress processing^22^. In the previous study, social evaluation and comparison of the participant’s performance were major contributing factors to the stress response. The right STG was associated with stress^20,21,39^ and attention processing^40^. Social components are important characteristics for the female self-representation^41^ and thus may lead to stronger engagement in tasks including social evaluation in women. This difference may explain the sex-specific patterns observed in the right STG in the current study compared to the previous task including social evaluation.

Regarding involvement of the insula, the specific part of the anterior insula points to reliving and processing emotions^42–44^. Therefore, the engagement of the insula in men may reflect reliving of emotions during the achievement stress task. Furthermore, increased precuneus/PCC and hippocampal activation in male participants suggest more pronounced self-related memory and self-referential processes compared to female participants^45–47^. This may reflect self-related thoughts about performance and recall of previous experiences of mental arithmetic challenges.

Higher activation in the cluster extending from the cerebellum to the fusiform gyrus may indicate mediation between remembering and imagining situations^48^. Thus, men may integrate past stressful experiences and future states of challenging situations during the achievement stress task, which may be seen as a potential stress coping mechanism.

Taken together, increased cortisol and testosterone responses, better performance and the observed neural activation differences suggest that men were more engaged in performing the achievement stress task. Given the lack of group differences in subjective ratings and in achievement motivation or coping scores, we can only speculate that our findings indicate that men put more effort in performing well and fulfilling expectations related to the self-concept of achievement and success^44^.

### The role of self-esteem in stress processing

Women and men showed similar self-esteem scores – although we had hypothesized otherwise, we believe this result to be representative of the distribution of self-esteem scores in student samples^11^. Moreover, we did not observe a significant interaction of sex-by-stress-by-self-esteem as we had hypothesized. Instead we observed a general effect of self-esteem on stress reactivity in women and men.

Activation of the precuneus/PCC and the insula were negatively associated with self-esteem. Insula activation was previously related to low self-esteem in a social stress task^10,49^. Assuming that the activation of the precuneus/PCC serves as an indicator for self-referential cognition, episodic memory retrieval, mind-wandering and self-generated thoughts^45,50^, and activation of the anterior insula as a reliving of emotions^42–44^, we interpret these associations to indicate that individuals with lower self-esteem have more self-related thoughts and relive subjective emotional scenes in challenging situations. Thus, low self-esteem may be associated with increased thinking about oneself and about past negative emotional experiences.

The right STG was previously associated with stress^20,21,39^ and attention processing^40^. It was also considered as an executive node during emotion regulation^51^. Stronger activation of the right STG in persons with lower self-esteem may point to stronger attention processing and emotion regulation during stress exposure. These individuals may have to put more effort in regulating the stressful experience, probably by increasing cognitive and emotion control through activation of the right STG, while performing the task – again not what we had expected apriori.

Low self-esteem may act as a vulnerability factor for psychiatric disorders such as depression or eating disorders^5^. Low self-esteem is associated with heightened stress reactions^13,14^, which in turn is dysfunctional in several clinical populations^52^. Moreover, women generally show lower global self-esteem than men^6,7^. The current results extend previous findings by indicating associations between neural stress reaction and self-esteem – though irrespective of sex. Thus, in women and men, a poor self-evaluation seems to have specific effects in stressful situations: Our data suggest that participants with lower self-esteem put more effort in cognitive performance control, stress and emotion regulation and, furthermore, think about themselves and relive previous stressful experiences during acute stress.

### Limitations

The current study has limitations that should be considered in future research: Removing the social aspects during the modified MIST-task may have reduced the stressfulness, leading to reduced effects on cortisol, although a similar effect has been reported previously^28,29^. Assessing saliva samples at only two time points may have masked variations in response peak. Furthermore, cortisol levels are reported to differ between follicular and luteal cycle phase in stressful situations^18,53,54^. Analyses of cycle effects are beyond the scope of the current manuscript but future studies may attempt to clarify interactions between stress, hormones and self-esteem across the menstrual cycle. Additionally, to assess mood changes following the stress induction, we only relied on the PANAS and ESR but did not explicitly ask how stressed participants felt or apply other measures probably more suitable to detect stress-related mood changes.

Moreover, as this is the first attempt to combine self-esteem with neural stress reactions in females and males by using an achievement stress task, it is unclear whether effects reported here will also be apparent in studies using different stress tasks, e.g., those favoring women.

## Conclusion

Sex differences in stress response emerged on the behavioral, hormonal and neural level. Hence, our findings suggest a stronger engagement in the achievement stress task in men than in women. Notably, self-esteem is a specific contributor to stress reactions in both sexes and is associated with activation in neural regions associated with attention, emotion and stress regulation irrespective of sex. Further research is needed to better understand the function of self-esteem in stress resilience. High levels of self-esteem are protective against adverse mental health outcomes and are important resilience factors^3,4^. Self-esteem and testosterone may enable individuals to cope more effectively with stressful events^3^. By clarifying the role of self-esteem in stress reactivity the current results are opening potential clinical avenues to protect people with low self-esteem against the negative impact of stress.

## Materials and Methods

### Sample

Eighty right-handed non-smoking Vienna University students (40 females) participated in this study (age: males: 24.4 years (SD = 3.4), females: 24.7 years (SD = 3.9); intelligence: males: IQ 103.8 (SD = 9.6), females: IQ 103.2 (SD = 10.2)). Participants were screened for the following exclusion criteria: history of neurological and psychiatric disorders (confirmed via structured clinical interview, SCID^55^), chronic illnesses, drug or hormone intake, working night shifts, engaging in competitive sports, recent or current pregnancy, premenstrual dysphoric disorder, allergic asthma and other common factors of MR-incompatibility. In order to control for menstrual cycle effects, half of the females were tested in mid-luteal phase (in a 28 day cycle: between days 18–23) and the other half during early follicular phase (days 1–5). This was controlled by documenting three previous cycles and the onset of the following menses and validated with progesterone and estradiol baseline levels. Women with atypical levels for the respective cycle phase were excluded from analysis. Written informed consent was obtained. The study was approved by the local Institutional Review Board of the Medical University of Vienna and participants were treated according to the Declaration of Helsinki (1964).

### Procedure

We used a modified version of the Montreal Imaging Stress Task (MIST)^19^ to induce achievement stress. As in the original version, participants were required to solve arithmetic problems. During control blocks, task difficulty was adapted to participants’ performance and accuracy feedback was provided. During stress blocks a time limit was set to restrict success rate to 20–45%. The task lasted for about 10 minutes. Two control blocks (70 seconds each) were followed by two stress blocks (70 seconds each); this sequence was repeated twice. Modification addressed the social aspects, which were excluded in this version: participants did not receive negative social feedback between runs and did not see their performance in comparison to performance of a peer-group. The paradigm was modified to exclude interaction effects of achievement stress and sex resulting from social evaluation and competition: The release of stress-related hormones was previously associated with social evaluation and exclusion^56,57^ as well as with competitive behavior^58,59^.

To examine the effect of the stress task on subjective distress, participants provided positive and negative affect ratings by means of the Positive and Negative Affect Scale (PANAS^24^) and the emotional self-rating (ESR^25^) before and after the MIST.

All participants performed another fMRI session on the same day, which was not related to the achievement stress task, and took place at least 60 minutes apart from the stress session. Results from this session will be presented elsewhere.

To assess self-esteem, we employed the Rosenberg-scale^60^. Participants responded to the 10-item self-report measure via a 5-point Likert scale (“strongly agree” – “strongly disagree”). Moreover, we assessed stress coping strategies (Coping Inventory for Stressful Situations, CISS^61^) and achievement motivation (Achievement Motivation Inventory, AMI^62^) to explore potential group differences.

All measurement sessions took place at the MR Center of Excellence at the Medical University of Vienna (Vienna, Austria), and were scheduled between 2:30 pm and 5:30 pm to control for circadian hormone rhythms. Participants were asked to refrain from exercise or alcohol consumption for 24 h prior to the session, medication, caffeine and drug intake on the test day, and food or drinks other than water for two hours before the session. Upon arrival, participants received detailed instructions and provided pre-stress saliva samples (T1) and subjective positive and negative mood. Post-stress saliva samples (T2) were taken 25 minutes after the onset of the stress condition.

Saliva samples were stored at −20 °C until shipping to the analysis laboratory (SwissHealthMed, Aying, Germany), where they were frozen at −20 °C over-night, then thawed and centrifuged. Competitive luminescence immunoassay kits (LUMI) were used to obtain cortisol, testosterone and progesterone concentrations. These kits have proven reliability and validity (testosterone: intra-assay coefficient of variability (CV) < 4% and inter-assay CV < 7%, cortisol: intra-assay CV < 4% and inter-assay CV < 5%, progesterone: inter-assay CV < 4% and inter-assay CV < 5%).

### FMRI data acquisition

Functional and anatomical data were acquired on a 3 T TIM Trio scanner (Siemens Medical Solutions, Erlangen, Germany) equipped with the manufacturer’s 32-channel head coil. Stimuli were projected onto a screen, which participants viewed via a mirror mounted on the head coil. We recorded 23 interleaved slices with a distortion-corrected gradient-echo EPI-sequence and the following imaging parameters: TE/TR = 38/1800ms, flip angle = 90°, voxel size = 1.5 × 1.5 × 3 mm, bandwidth = 1446 Hz/pixel, 1.8 mm slice gap. Additionally, a high-resolution anatomical image using an MPRAGE sequence (3-D Magnetization Prepared Rapid Gradient Echo) was acquired from every participant.

### Statistical Analysis

#### Behavioral and hormone data

Statistical analysis of behavioral data was performed using SPSS 20.0 (SPSS Statistics, Version 20.0, IBM, USA). Hormone data (cortisol/testosterone/progesterone) were log-transformed (y = log10(x + 1)) prior to statistical analyses and further analyzed with sex-by-time ANOVAs with repeated measures. Similar ANOVAs were used to analyze sex differences in performance (error-ratio, i.e. number of incorrect or no response trials divided by number of processed trials) in the stress task (factors: sex and condition) and in subjective ratings (factors: sex and time), while questionnaire data (self-esteem, CISS, AMI) were analyzed using independent samples t-tests.

#### FMRI data

Preprocessing and analyses of the imaging data were performed with statistical parametric mapping (SPM8, Wellcome Department of Imaging Neuroscience, London) implemented in Matlab R2015b (Mathworks Inc., Sherborn, MA, USA) using standard algorithms and parameters unless specified differently. Images were realigned to correct for head movement, slice-time corrected^63^, spatially normalized to MNI (Montreal Neurological Institute) stereotactic space using unified segmentation and finally smoothed with an 8 mm full-width-at-half-maximum Gaussian kernel. Pre-processed data were analyzed using a general linear model with three conditions (stress, control, rest), which were modeled with separate regressors convolved with the canonical hemodynamic response function. Additional nuisance regressors included realignment parameters and potentially confounding signals from white matter and ventricles. For each participant, main effects were computed by applying appropriate baseline contrasts (simple effects) for each condition. These first-level individual contrasts were then fed into the second-level group analysis using a flexible factorial model (factors: condition, subject, sex). All functional findings are reported at a cluster-level FWE corrected threshold of p < 0.05 (cluster-forming threshold at voxel-level p < 0.001).

To better characterize the observed whole-brain sex differences and to test for associations between neural activation and self-esteem scores, we performed linear regression analyses between self-esteem, hormones and neural activation as the output variable in specific regions of interest (ROIs). We specified these ROIs based on previous reports on sex differences in neural stress responses and self-esteem^2,21^: thus, we chose right insula and right STG. These functional ROIs were extracted based on statistically significant regions emerging in the contrasts of the current study. The mean values of a 10 mm sphere at specific MNI coordinates were extracted for the functional ROIs for each participant with SPM8 (right insula [x: 38, y: 26, z: −4]; right STG [x: 62, y: −44, z: 16]).

Linear regression analyses (hierarchical entry) with the predictors sex, self-esteem, and each hormone separately (testosterone, progesterone or cortisol), as well as their interaction terms, and the brain activation of the two ROIs during stress as outcome variable, were performed. Results of these analyses were corrected for multiple comparisons.
